# Study on dose-dependent, frequency-dependent, and accumulative effects of 1.5 GHz and 2.856 GHz microwave on cognitive functions in Wistar rats

**DOI:** 10.1038/s41598-017-11420-9

**Published:** 2017-09-07

**Authors:** Shengzhi Tan, Hui Wang, Xinping Xu, Li Zhao, Jing Zhang, Ji Dong, Binwei Yao, Haoyu Wang, Hongmei Zhou, Yabing Gao, Ruiyun Peng

**Affiliations:** 1 Department of Experimental Pathology, Beijing Institute of Radiation Medicine, Beijing, P. R. China; 2 Division of Radiation Protection and Health Physics, Beijing Institute of Radiation Medicine, Beijing, P. R. China

## Abstract

Many studies have revealed the cognitive decline induced by microwave radiation. However, the systematic study on dose-dependent, frequency-dependent and accumulative effects of microwave exposure at different frequencies was lacking. Here, we studied the relationship between the effects and the power and frequency of microwave and analyzed the accumulative effects of two different frequency microwaves with the same average power density. After microwave radiation, declines in spatial learning and memory and fluctuations of brain electric activities were found in the 10 mW/cm^2^ single frequency exposure groups and accumulative exposure groups. Meanwhile, morphological evidences in hippocampus also supported the cognitive dysfunction. Moreover, the decrease of Nissl contents in neurons indicated protein-based metabolic disorders in neurons. By detecting the key functional proteins of cholinergic transmitter metabolism, cytokines, energy metabolism and oxidative stress in the hippocampus, we found that microwave could lead to multiple metabolic disorders. Our results showed that microwave-induced cognitive decline was largely determined by its power rather than frequency. Injury effects were also found in accumulative exposure groups. We particularly concerned about the safety dose, injury effects and accumulative effects of microwaves, which might be very valuable in the future.

## Introduction

In the past century, microwaves have been widely used in many aspects, such as communication, industry, medicine and military^1–3^. As a kind of nonionizing radiation, the potential health hazards of microwave have been getting more and more attentions. In recent decades, electromagnetic waves have been considered as a new form of environmental pollution by the World Health Organization *(WHO)* and classified as “possibly carcinogenic to humans” *(Group 2B)* by the International Agency for Research on Cancer *(IARC)* ^4^. The relevant safety standards *(C95.1)* were established by the Institute of Electrical and Electronics Engineers *(IEEE)*.

**Table 1:** Groups and Microwave Exposure.

Groups	Average power density of 2.856 GHz microwave	Average power density of 1.5 GHz microwave	SAR***(W/kg)***
C*	0	0	0
S5	5 mW/cm^2^ for 6 min	0	1.7
L5	0	5 mW/cm^2^ for 6 min	1.8
SL5^▵^	5 mW/cm^2^ for 6 min	5 mW/cm^2^ for 6 min	1.7 for the first 6 min +1.8 for the last 6 min
S10	10 mW/cm^2^ for 6 min	0	3.3
L10	0	10 mW/cm^2^ for 6 min	3.7
SL10^▵^	10 mW/cm^2^ for 6 min	10 mW/cm^2^ for 6 min	3.3 for the first 6 min +3.7 for the last 6 min

^*^The animals in the control group were placed in the polypropylene cages for 6 min.

^▵^The rats in the SL5 and SL10 groups were firstly exposed the 2.856 GHz microwave for 6 min and then were immediately exposed to the 1.5 GHz microwave for 6 min.

It was reported that microwave could induce the impairment in learning and memory functions, brain electric activities, and brain structures^5–7^. Besides, many studies were made to test the health hazards of microwave and the nervous system was believed very sensitive to microwave exposure^8^. Study showed that the microwaves induced biological effects were related with their power densities, frequencies, waveforms, modulation and durations of exposure^9^. Effects caused by different kinds of microwave might be different. However, microwaves in the experimental studies were mostly of single frequency, the frequency-dependent effects had never been talked. In our studies, the frequency-dependent effects between 2.856 GHz and 1.5 GHz at same power density and the does-dependent effects between 5 mW/cm^2^ and 10 mW/cm^2^ at same frequency were studied.

However, microwaves in the experimental studies were mostly of single frequency, while the real environment was usually consisted of many different frequency or power microwaves. The accumulative effects of different microwaves were ignored.

Some studies had studied the accumulative effects of various communication microwaves (849 MHz, 1.95 GHz) and found no significant changes, while the power of communication microwaves was very low^1, 10–12^. There were few studies about the accumulative effects of high power microwaves. In our study, the accumulative effects of 2.856 GHz microwave exposure and 1.5 GHz microwave exposure were studied.

Besides, it was proved that the microwave exposure could relieve injuries caused by ionizing radiation^13–15^, which indicated that the biological effects of two different electromagnetic waves could not be simply evaluated by the exposure power. The possible interaction effects between 2.856 GHz and 1.5 GHz should be considered.

Both of 2.856 GHz and 1.5 GHz microwaves were common used in daily life. In this study, animals were treated by the single 2.856 GHz microwave, single 1.5 GHz microwave and accumulative 2.856 GHz and 1.5 GHz microwave. The levels of average power densities were set as 5 mW/cm^2^ and 10 mW/cm^2^. The power of 5 mW/cm^2^ was considered safe for human according to safety standard issued by IEEE. In animal experiments, the power of 5 mW/cm^2^ was also found safe for Wistar rats^5, 16^, which were the same animal strain used in this paper. After microwave radiation, we detected whether microwave could produce changes in spatial learning and memory abilities, EEGs and hippocampal morphology. Meanwhile, the Nissl substance in hippocampal neurons and related neuronal metabolism marks were detected to consider its relationship with the cognition function. The metabolism markers, acetylcholinesterase (AchE), brain derived neurotrophic factor (BDNF), cytochrome C oxidase (COX) and superoxide dismutase (SOD), were selected for considering their roles in cholinergic transmitter metabolism, neural cytokines, neural energy metabolism and neural oxidative stress.

**Figure 1:**
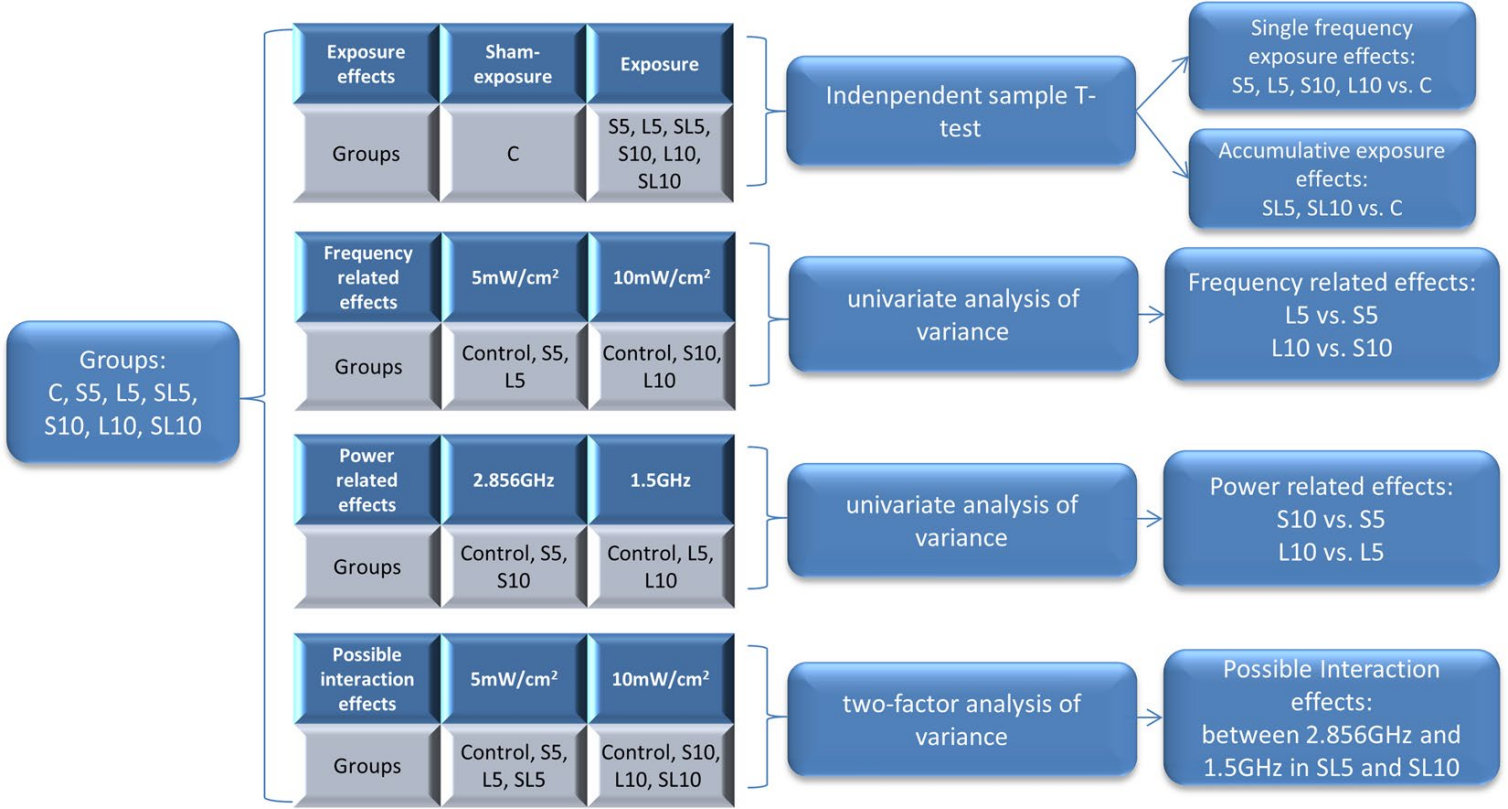
Experiment design and statistical methods.

The experimental design could be divided into four kinds, which would be introduced in “Materials and Methods” part in detail (Fig. 1). The first design was to evaluate the exposure effects (single frequency effects and accumulative effects) of 2.856 GHz and 1.5 GHz microwave in exposure groups. The second design of this study was to evaluate the frequency-dependent effects between 2.856 GHz and 1.5 GHz microwave. The third design was to evaluate the does-dependent effects between 5 mW/cm^2^ and 10 mW/cm^2^ microwave. The fourth design was to evaluate the possible interaction effects between 2.856 GHz and 1.5 GHz microwave exposure.

## Materials and Methods

### Ethics statement

Animal work in this study was approved by the Beijing Institute of Radiation Medicine Animal Care and Use Committee. It was carried out on the basis of the National Institute of Health Guide for the Care and Use of Laboratory Animals.

### Experimental animals and groups

175 Male Wistar Rats (weights 200 ± 20 g) were provided by the Laboratory Animal Center, Beijing Institute of Radiation Medicine, Beijing, China. Rats were maintained at a constant temperature (22 ± 1 °C) and relative humidity (60%) under a regular dark-light schedule (lights on from 7 a.m. to 7 p.m.).

The 2.856 GHz and 1.5 GHz microwaves belong to S-band and L-band respectively and the power densities used in our study were 5 mW/cm^2^ and 10 mW/cm^2^. Therefore, for convenience, we used the character “S” to represent the exposure group of 2.856 GHz, and the character “L” to represent the exposure group of 1.5 GHz. Meanwhile, numbers “5” and “10” were used to represent the power densities for each group.

The rats were randomly divided into 7 groups (n = 25 per group): (1) the control group (C group); (2) the 5 mW/cm^2^ microwave exposure group at the frequency of 2.856 GHz (S5 group); (3) the 5 mW/cm^2^ microwave exposure group at the frequency of 1.5 GHz (L5 group); (4) the 10 mW/cm^2^ microwave exposure group at the frequency of 2.856 GHz (S10 group); (5) the 10 mW/cm^2^ microwave exposure group at the frequency of 1.5 GHz (L10 group); (6) the 5 mW/cm^2^ accumulative microwave exposure group (SL5 group); (7) the 10 mW/cm^2^ accumulative microwave exposure group (SL10 group) (Table 1).

### Microwave exposure system and dosimetry

Two microwave sources generating pulsed microwaves at the frequency of 2.856 GHz and 1.5 GHz respectively were used in this study. The exposure system and animal placement were same to Hui’s study^5^. The two microwave sources were placed beside each other in an electromagnetic shield chamber. In accumulative exposures, rats were firstly exposed under the 2.856 GHz antenna, then the rats were parallel moved to under the 1.5 GHz antenna by a conveyor belt. Interval time between the two exposures was very short and negligible.

The specific absorption rate *(SAR)* of rats could be affected by various variability factors^17^. In this paper, the SAR of microwave exposure was calculated based on the exposure scenario in each group. The SAR calculation was based on the finite difference time domain *(FDTD)* method and the formula: $SAR=\sigma {E}^{2}/\rho$(W/kg)^18^. In the formula, E is the electric field strength *(V/m)*, σ is the electric conductivity *(S/m)*, and ρ refers to the sample density *(kg/m* ^3^). In our study, the software for calculating SAR in our study was the simulation platform Empire: IMST-Empire v-4.10 *(GmbH, Kamp-Lintfort, Germany)*. The SAR for the seven groups were calculated and described in Table 1.

### Temperature monitoring

The supernatant temperatures of experimental animals were monitored using an infrared temperature sensor *(FLIR A*4*0, USA)* before and immediately after microwave exposure.

### Morris water maze (MWM)

The MWM task was performed in a circular pool *(1*5*0 cm in diameter)* filled with water and maintained at 23 ± 0.5 °C in a special room at constant temperature, humidity and brightness. The surface of the escape platform *(12 cm in diameter)* was submerged 1.5 cm below the water surface at a specific location for the entire session. The pool was surrounded by thick curtains to hide extra-maze visual cues from the rats. Rats were trained to find the submerged escape platform during four consecutive daily sessions. Each session consisted of four trials. Four different starting positions, equally spaced around the perimeter of the pool, were used in a fixed order. At 1d, 2d, 7d, 14d, and 28d after microwave exposure, the spatial learning and memory was assessed. Rats behavior in the MWM experiments during the training and behavior test procedures was digitally recorded using a SLY-MW system *(Beijing Sunny Instrument Co., China)* and the average escape latency *(AEL)* was analyzed.

### EEG recording

At 7d after microwave exposure, 5 rats in each group were evaluated under light anesthesia conditions using a four-electrode configuration. The EEG recorded the collective activity of neurons through electrodes placed on the surface of the scalp. The EEG signals were obtained through a BIOPACMP-150 system *(USA)* and power spectral analyses were performed on spontaneous EEG segments.

### Analysis of hippocampal morphology

#### Microstructure examination

At 7d after microwave exposure, 5 rats in each group were sacrificed humanely for pathological examination. Brains were removed and fixed in 10% buffered formalin solution. Tissue containing the hippocampus were embedded in paraffin and cut at 5 μm thick in the coronal plane. The sections were then stained with hematoxylin-eosin *(H&E)* and observed blindly under a light microscope *(Leica DM6000, Leica, Wetzlar, Germany)* for microstructure examination.

#### Ultrastructure examination

The hippocampal specimens *(1mm³)* were dissected from the DG area at 7d after microwave exposure. Briefly, the samples were placed in 2.5% glutaraldehyde and post-fixed with 1% osmium tetroxide. After graded ethyl alcohols, the cubes were embedded in EPON618. Thin sections laid on copper mesh were stained with heavy metals, uranyl acetate, and lead citrate for contrast^19^. A Hitachi-H7650 transmission electron microscope *(TEM, Hitachi, Japan)* was used to observe the hippocampal ultrastructure.

### Nissl body examination

The prepared sections of each group were then stained with Toluidine blue and observed blindly under a light microscope *(Leica DM6000, Leica, Wetzlar, Germany)* for Nissl body examination at 7d after microwave exposure.

### Immunohistochemical staining (IHC)

Paraffin-embedded brain sections of each group at 7d after microwave exposure were used for immunohistochemistry: (1) AchE was detected by mouse sourced AchE antibody *(Novus Biologicals, Colorado,USA)*; (2) BDNF was detected by rabbit sourced BDNF antibody *(Abcam, Cambridge, UK)*; (3) COX was detected by rabbit sourced COX antibody *(Cell Signaling Technology, Massachusetts, USA)*; (4) SOD was detected by rabbit sourced SOD antibody *(Cell Signaling Technology, Massachusetts, USA)*. Then, the sections were treated with horseradish peroxidase conjugated goat sourced mouse or rabbit antibody. Tissue sections were then colorized with diaminobenzidine *(DAB) (ZSGB, Beijing, China)*. The positive tissue sections were analyzed with CMIAS pathological image analysis system *(Beijing University of Aeronautics and Astronautics, China)*.

### Statistical analysis

Statistical analyses were carried out using SPSS 19.0 software.

The independent sample T-test was used to analyze the exposure effects between exposure groups and control group, including single frequency exposure effects and accumulative exposure effects. The univariate analysis of variance was used to analyze the frequency-dependent effects and power-dependent effects. The two-factor analysis of variance was used to examine the possible interaction effects between 2.856 GHz and 1.5 GHz microwave in accumulative exposure groups (Fig. 1).

The accepted level of significance for all tests was p < 0.05. According to the comparison target and meanings, significance markers were classified as follows: significant exposure effects, ^*^P < 0.05, ^**^P < 0.01, *(vs. C)*; significant frequency-dependent effects, ^▵^P < 0.05, ^▵▵^P < 0.01 *(L10 vs. S10 and L*5 *vs. S5)*; significant does-dependent effects, ^#^P < 0.05, ^##^P < 0.01 *(S10 vs. S5 and L10 vs. L5)*; significant interaction effects, ^★^P < 0.05, ^★★^P < 0.01.

## Results

### Temperatures increased less than 1 °C

We measured the rats’ surface temperatures (*n* = *4*) before and immediately after microwave exposure by infrared temperature sensor. According to the results, temperatures increased less than 1 °C after microwave exposure (Table 2), indicated that the thermal effects could be compensated by the physiological temperature regulation of organism and the effects discussed in this paper were basically non-thermal effects.

**Table 2:** Body surface temperatures before and immediately after microwave exposure.

Groups	Body surface temperatures before microwave exposure (°C)	Body surface temperatures after microwave exposure (°C)
C	36.35 ± 0.24	36.63 ± 0.39
S5	36.60 ± 0.26	36.80 ± 0.14
L5	36.75 ± 0.26	36.78 ± 0.33
SL5	36.68 ± 0.25	36.83 ± 0.22
S10	36.45 ± 0.24	36.75 ± 0.39
L10	36.63 ± 0.15	36.78 ± 0.17
SL10	36.93 ± 0.19	36.65 ± 0.34

### Spatial learning and memory ability of rats declined after microwave exposure

#### AELs prolonged after 2.856 GHz and 1.5 GHz microwave exposure

**Figure 2:**
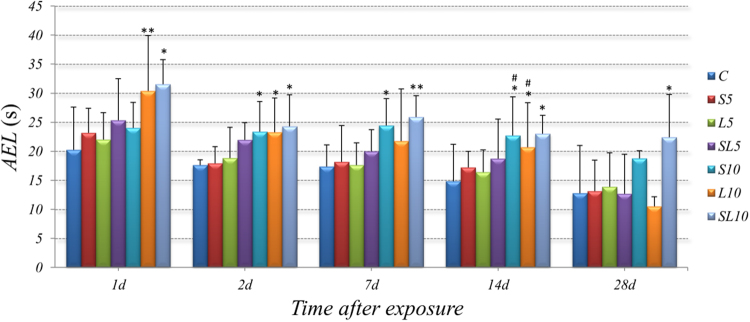
Microwave exposure prolonged AELs in rats, data were shown as mean ± SD. ^*^P < 0.05, ^**^P < 0.01, vs. control; ^#^P < 0.05, S10 vs. S5 & L10 vs. L5.

The MWM was performed at 1d, 2d, 7d, 14d, and 28d after microwave exposure. AEL was the time for rats to find the platform. As shown in Fig. 2, compared with control group: (1) the single or accumulative 5 mW/cm^2^ microwave exposure *(S5, L5, SL5)* caused no significant changes in AELs; (2) the single 10 mW/cm^2^ 2.856 GHz microwave exposure caused significantly prolonged AELs at 2d *(p* = *0.016, n* = *15)*, 7d *(p* = *0.011, n* = *15)*, and 14d *(p* = *0.033, n* = *15)* after microwave exposure; (3) the single 10 mW/cm^2^ 1.5 GHz microwave exposure significantly prolonged AELs at 1d *(p* = *0.010, n* = *15)*, 2d *(p* = *0.036, n* = *15) and* 14d *(p* = *0.013, n* = *15)* after microwave exposure; (4) the 10 mW/cm^2^ accumulative exposure significantly prolonged AELs at 1d *(p* = *0.005, n* = *15)*, 2d *(p* = *0.01*2*, n* = *15)*, 7d *(p* = *0.007, n* = *15)*, 14d *(p* = *0.018, n* = *15)* and 28d *(p* = *0.0*2*2, n* = *15)* after microwave exposure.

#### No frequency-dependent effect between 2.856 GHz and 1.5 GHz in AELs

In order to figure out the frequency-dependent effects of 2.856 GHz and 1.5 GHz microwave, the results from groups of same average power density but with different frequency were compared and statistically analyzed *(L5 vs. S5, L10 vs. S10)*. There were no significant differences of AEL between the 2.856 GHz microwave exposed rats and the 1.5 GH*z* microwave exposed rats (Fig. 2).

#### Significant does-dependent effects of 2.856 GHz and 1.5 GHz microwave in AELs

Groups of same frequency with different power density were compared and statistically analyzed to figure out the does-dependent effects *(S10 vs. S5, L10 vs. L5)*. Results found that: (1) Rats in the *S10* group showed significantly prolonged AELs than that of *S5* at 1d *(p* = *0.041, n* = *15)* and 14d after microwave exposure *(p* = *0.013, n* = *15)*; (2) Significantly prolonged AELs were found in the *L10* group when comparing to the *L5* at 14d after microwave exposure *(p* = *0.024, n* = *15)* (Fig. 2).

#### No interaction effect between 2.856 GHz and 1.5 GHz microwave in AELs

According to the statistical analysis, no significant interactions were found between the 2.856 GHz and 1.5 GHz microwave exposure groups (Fig. 2).

### Alterations of electroencephalography (EEG) after microwave exposure

#### 2.856 GHz and 1.5 GHz microwave induced fluctuations in EEGs

**Figure 3:**
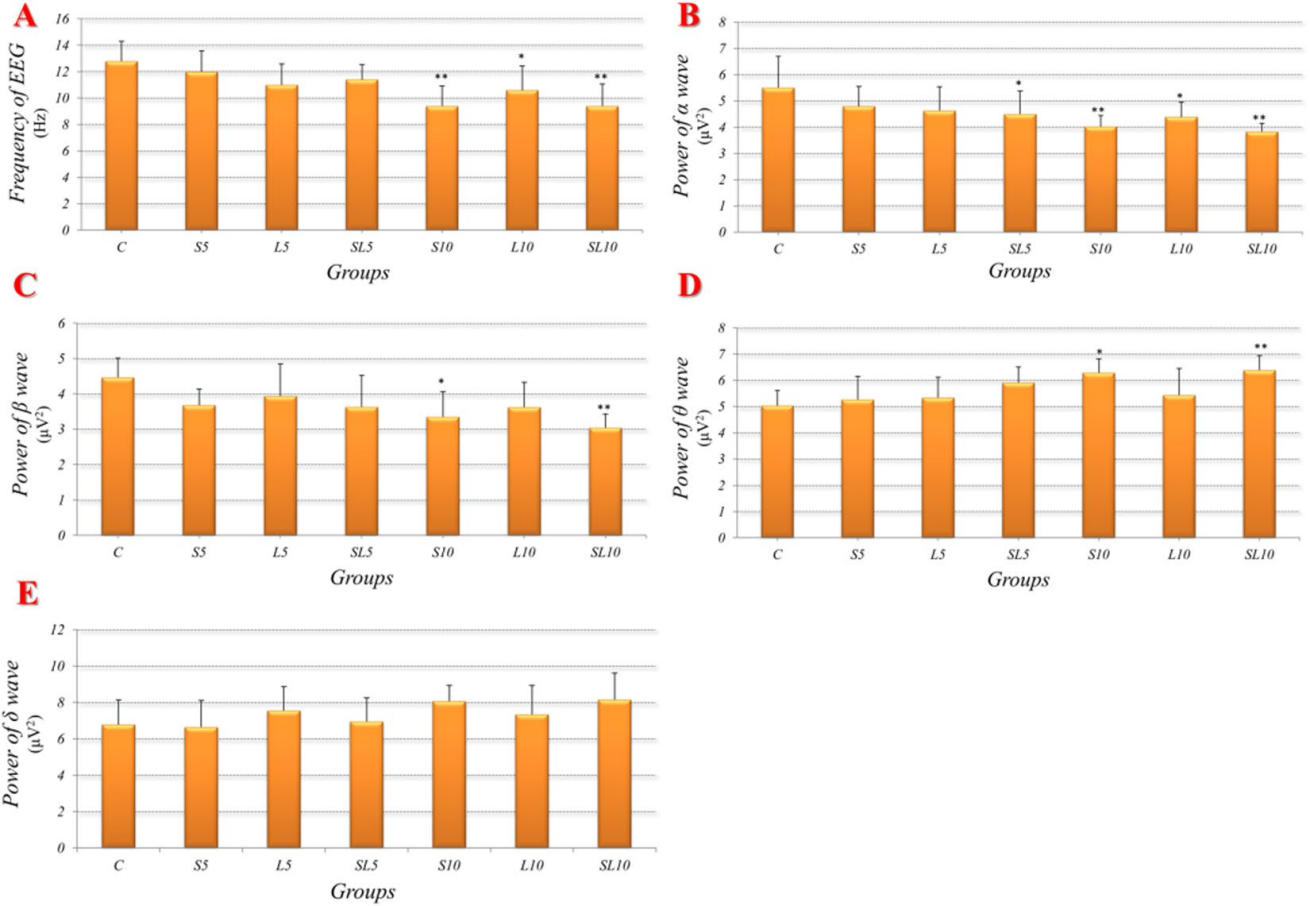
Alterations of electroencephalography (EEG) in rats at 7d after microwave exposure, changes in frequencies of EEG (**A**) and powers of α, β, θ, δ waves (**B–E**) were displayed in the form of charts. Data were shown as mean ± SD. *P < 0.05, **P < 0.01, vs. control.

The frequency of EEG is an integrated reflection of 4 kinds of brain wave: α *(12–30 Hz)*, β *(8–12 Hz)*, θ *(4–8 Hz)*, δ *(1–4 Hz)*. The frequencies of EEG and the powers of α, β, θ, δ waves in each group were recorded at 7d after microwave exposure. As shown in Fig. 3, in comparison with the control group: (1) the single 5 mW/cm^2^ microwave exposure *(S5, L5)* did not cause any changes in EEG; (2) the accumulative 5 mW/cm^2^ microwave exposure *(SL5)* significantly reduced power of α wave *(p* = *0.049, n* = *5)*; (3) the single 10 mW/cm^2^ 2.856 GHz microwave exposure *(L10)* significantly reduced the EEG frequency *(p* = *0.002, n* = *5)* and power of α wave *(p* = *0.005, n* = *5)* and β wave (*p* = *0.015, n* = *5*) and significantly increased power of θ wave *(p* = *0.011, n* = *5)*; (4) the single 10 mW/cm^2^ 1.5 GHz microwave exposure *(L10)* significantly reduced the EEG frequency *(p* = *0.033, n* = *5)* and power of α wave *(p* = *0.030, n* = *5)*; (5) the accumulative 10 mW/cm^2^ microwave exposure *(SL10)* significantly reduced the EEG frequency *(p* = *0.002, n* = *5)* and power of α wave *(p* = *0.002, n* = *5)* and β wave *(p* = *0.002, n* = *5)* and significantly increased power of θ wave *(p* = *0.006, n* = *5)*. No significant differences were found in δ wave after microwave exposure when compared to the control group.

#### No frequency-dependent effects between 2.856 GHz and 1.5 GHz in EEGs

The groups of same exposure power density but with different frequencies were picked out and compared *(L5 vs. S5, L10 vs. S10)*. No significant differences were found in EEGs between the 2.856 GHz exposed groups and the 1.5 GHz exposed groups (Fig. 3).

#### No does-dependent effects of 2.856 GHz and 1.5 GHz microwave in EEGs

The groups of same frequency but with different power densities were picked out and compared *(S10 vs. S5, L10 vs. L5)*. No significant differences were found in EEG between the 10 mW/cm^2^ and 5 mW/cm^2^ microwave exposed groups (Fig. 3).

#### No interaction effects between 2.856 GHz and 1.5 GHz microwave in EEGs

According to the statistical analysis, no significant interactions were found between the 2.856 GHz and 1.5 GHz microwave exposure groups (Fig. 3).

### Pathological changes of hippocampus caused by microwave exposure

#### Microwave exposure caused microstructural changes in hippocampus

**Figure 4:**
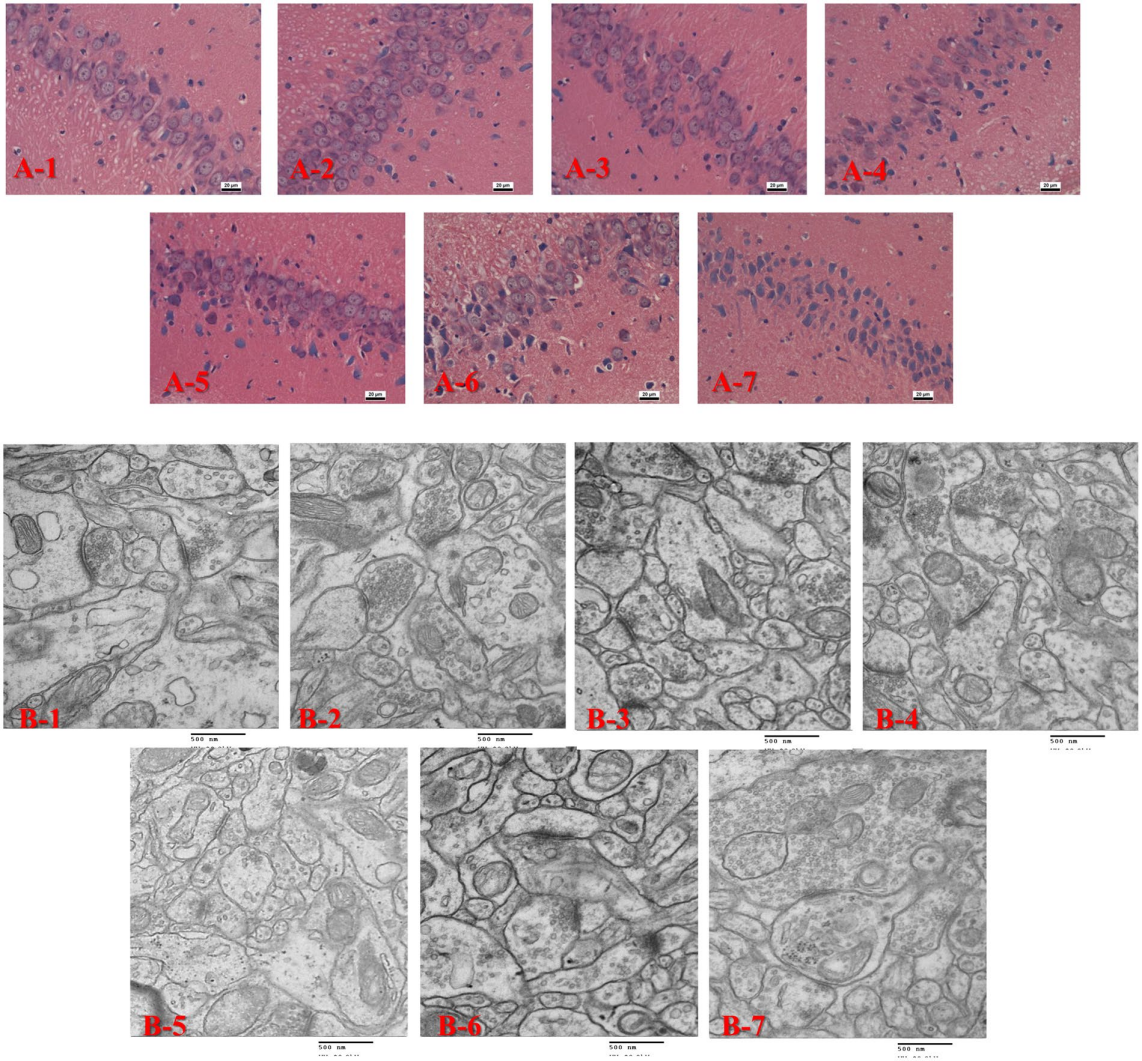
Pathological injuries and ultrastructure changes in hippocampus of rats at 7d after 2.856 GHz and 1.5 GHz microwave exposures, the microstructures were detected by LM at 7d after exposure to support the behavior results. A1–A3: Control group, S5 group and L5 group, and there was no degeneration of the neurons. A4–A7: SL5 group, S10 group, L10 group and SL10 group, injury changes such as karyopyknosis, irregular arrangement, cell edema, and broadening pericellular space were occurred. The heaviest injuries were occurred in SL10 group. B1–B3: Control group, S5 group and L5 group, and there was no degeneration of the synapse. B4–B7: SL5 group, S10 group, L10 group and SL10 group respectively, the synapse ultrastructure damages were observed in SL5 group (B4), S10 group (B5), L10 group (B6) and SL10 group (B7). (H&E scale bar = 20 μm, TEM scale bar = 500 nm).

In order to assess the morphological changes in hippocampus which were related with the cognitive abilities and brain electric activities, histological examinations of hippocampus were carried out. In each group, 5 brains containing hippocampus that fixed by formalin at 7d after microwave exposure were observed by H&E staining. Compared with the control group, no obvious changes were found in the S5 and L5 groups, but obvious injuries were observed in the SL5 group and the S10, L10, SL10 groups (Fig. 4 A1–A7). Injury changes such as karyopyknosis, irregular arrangement, cell edema, and broadening pericellular space were distributed in DG, CA1 and CA3 region. Moreover, changes in DG region were the most significant. The most serious injured group was considered as the SL10 group.

#### Microwave exposure caused ultrastructure changes in hippocampus

The effects of microwave radiation on the hippocampal ultrastructure were examined by TEM at 7d after microwave exposure (Fig. 4B1–B7). Compared with the control group, no obvious changes of ultrastructure were found in the S5 and L5 groups, but the obvious injuries were observed in the SL5 group and the S10, L10, SL10 groups. The injured neurons showed cytoplasmic relaxation, mitochondrial swelling and ridge rupture, even cavitation, rough endoplasmmicreticulum degranulation and swelling, broadening of the nuclear membrane gap, and concentration and margination of the chromatin. Injuries in the SL10 group were considered to be most serious.

### Nissl substance reduced in neurons after microwave exposure

#### 2.856 GHz and 1.5 GHz microwave exposure reduced the content of Nissl substance

**Figure 5:**
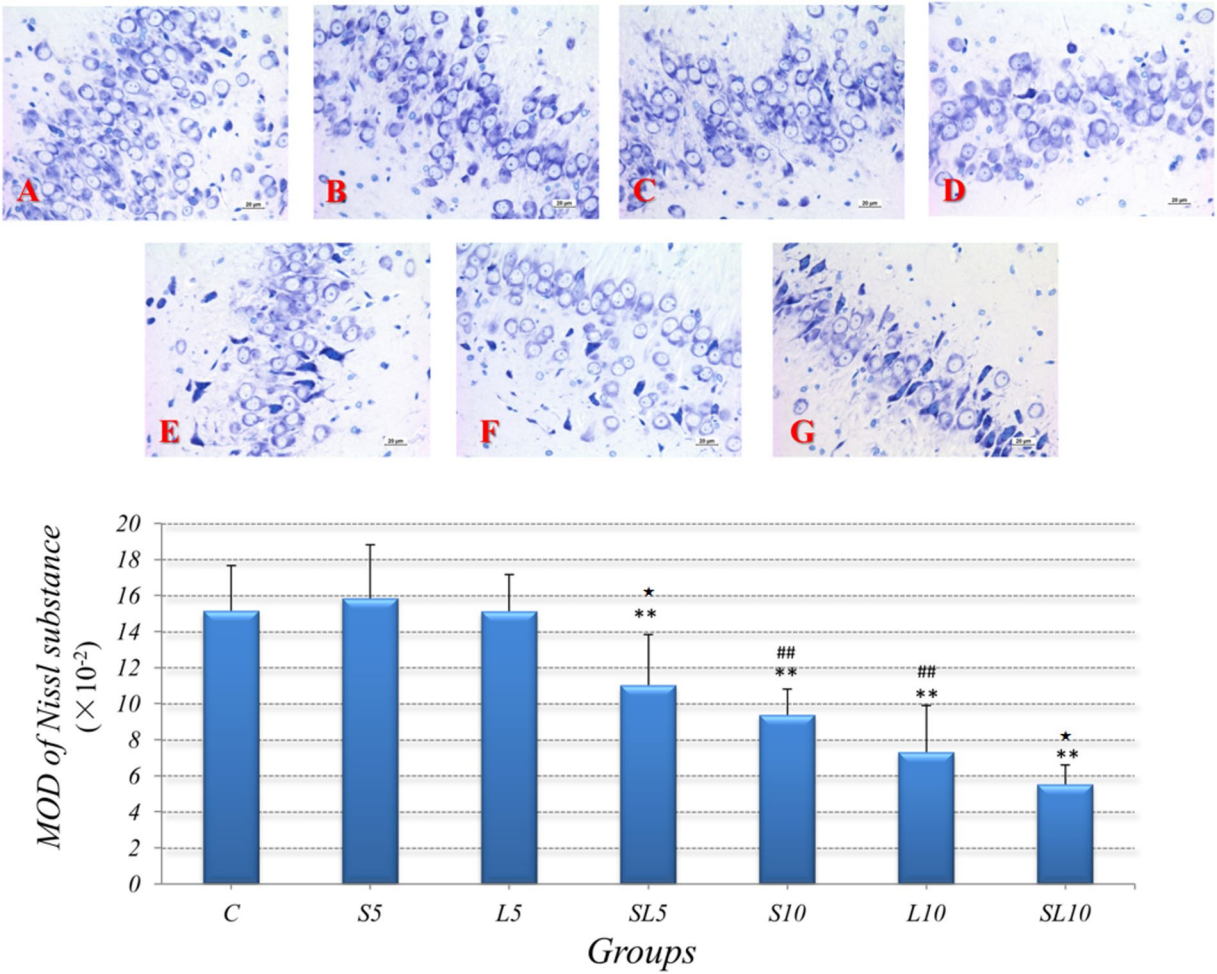
The content of Nissl substance reduced after 2.856 GHz and 1.5 GHz microwave exposure, (**A–C**) Control group, S5 group and L5 group, and the Nissl substance content were normal. (**D–G**) SL5 group, S10 group, L10 group and SL10 group, compared with the normal Toluidine blue staining in the control group (**A**), the reduced content of Nissl substance were observed in SL5 group (**D**), S10 group (**E**), L10 group (**F**) and SL10 group (**G**). (Toluidine blue staining scale bar = 20 μm). Quantized data were shown as mean ± SD. ^**^P < 0.01, vs. control; ^##^P < 0.01, S10 vs. S5 & L10 vs. L5; ^★^P < 0.05, possible interaction effects between 2.856 GHz and 1.5 GHz exposure.

Figure 5 showed the toluidine blue staining of Nissl substance in hippocampus, which were then quantitatively analyzed by MOD. The Nissl substances were dyed deep blue and existed in the cytoplasm of the neurons.

Based on the quantitative analysis, no significant differences were found between the single 5 mW/cm^2^ groups *(S5, L5, SL5)* and the control group. In comparison with the control group, the contents of Nissl substances in accumulative 5 mW/cm^2^ group *(SL5, p* = *0.002, n* = *5)*, single 10 mW/cm^2^ group (*S10, p* = *0.000, n* = *5; L10, p* = *0.001, n* = *5*) and accumulative 10 mW/cm^2^ group *(SL10, p* = *0.000, n* = *5)* significantly reduced.

#### No frequency-dependent effects between 2.856 GHz and 1.5 GHz microwave in the content of Nissl substance

We compared the results of quantitative analysis from groups of same power density but different frequencies to figure out the frequency-dependent effects, no significant differences were found between the 2.856 GHz and 1.5 GHz microwave exposed groups (Fig. 5).

#### Significant does-dependent effects of 2.856 GHz and 1.5 GHz microwave in the content of Nissl substance

We compared groups of same frequency but different power densities to figure out the does-dependent effects. The 10 mW/cm^2^ microwave significantly reduced contents of Nissl substances when compared to the 5 mW/cm^2^ groups *(S10 vs. S5, p* = *0.001, n* = *5; L10 vs. L5, p* = *0.002, n* = *5)*. Higher power density could cause more serious reduction in the content of Nissl substance (Fig. 5).

#### Possible aggravated interaction effects between 2.856 GHz and 1.5 GHz microwave in the content of Nissl substance

Possible significant interaction effects was found in 5 mW/cm^2^ and 10 mW/cm^2^ accumulative exposure groups *(5 mW/cm* ^2^ *: p* = *0.024, n* = *5; 10 mW/cm* ^2^ *: p* = *0.038, n* = *5)* (Fig. 5).

### Abnormal metabolism in hippocampus after microwave exposure

#### 2.856 GHz and 1.5 GHz microwave exposure reduced the expressions of AchE, BDNF, COX and SOD

**Figure 6:**
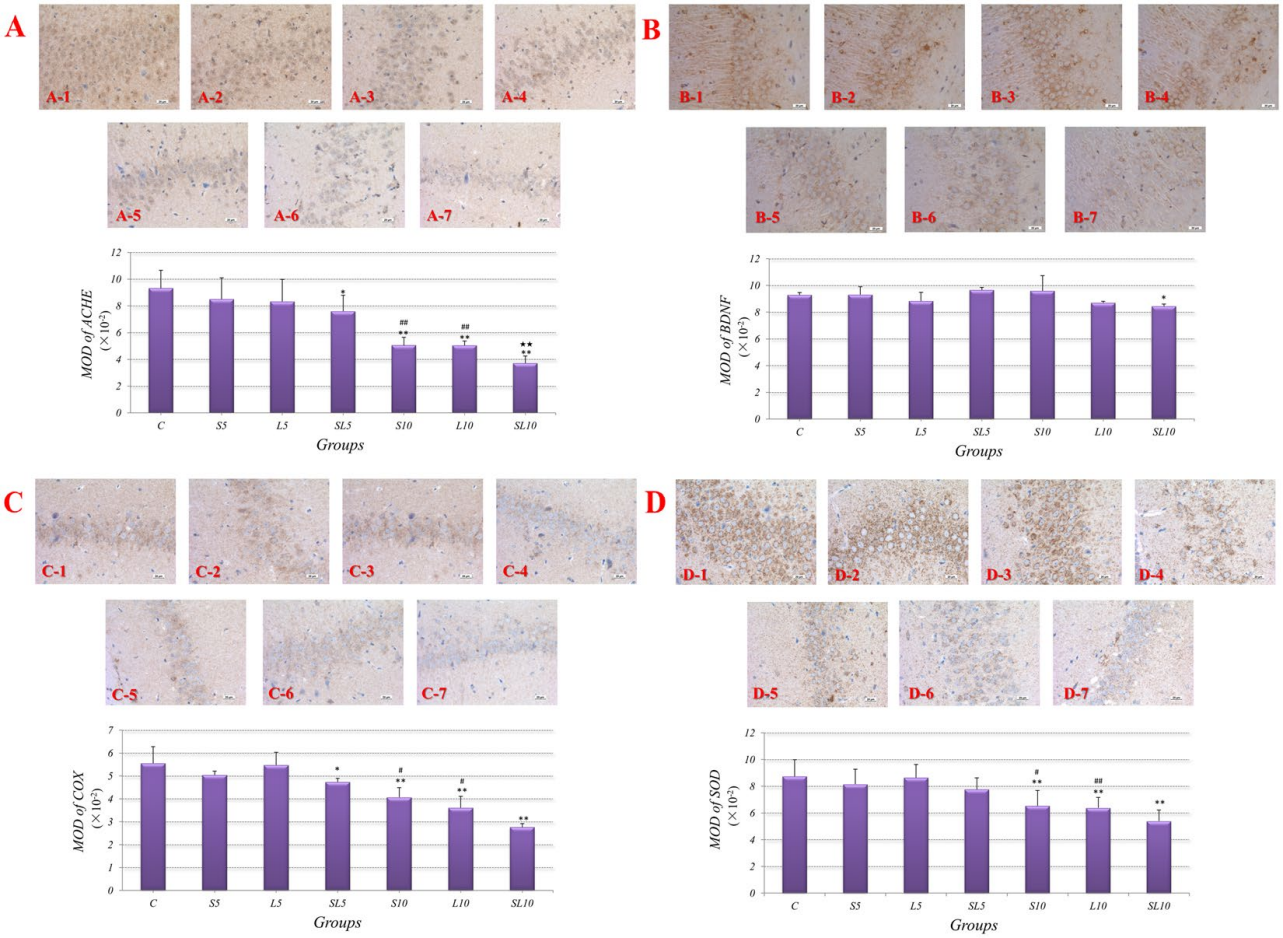
The expressions of AchE, BDNF, COX and SOD in hippocampus of rats were reduced at 7d after 2.856 GHz and 1.5 GHz microwave exposure. In section A, B, C and D, pictures 1 to 7 were control, S5, L5, SL5, S10, L10 and SL10 group. In S5 and L5 groups, the expression levels of AchE, BDNF, COX and SOD were similar compared with the control group; In SL5, groups, expression levels of AchE and COX were reduced compared with the control groups (A-4, C-4); In S10 groups, expression levels of AchE, COX and SOD were reduced compared with the control groups, and expression levels of AchE, COX and SOD were reduced compared with the S5 groups (A-5, C-5, D-5); In L10 groups, expression levels of AchE, COX and SOD were reduced compared with the control groups, and expression levels of AchE, COX and SOD were reduced compared with the L5 groups (A-6, C-6, D-6); In SL10 groups, expression levels of AchE, BDNF, COX and SOD were reduced compared with the control groups, and expression levels of COX were reduced compared with the S10 and L10 groups. (IHC scale bar = 20 μm), MOD values analyzed from immunohistochemistry images were shown as mean ± SD. *P < 0.05, **P < 0.01, vs. control; ^#^P < 0.05, ^##^P < 0.01, S10 vs. S5 & L10 vs. L5; ^★★^P < 0.01, possible interaction effects between 2.856 GHz and 1.5 GHz exposure.

Figure 6 showed the immunohistochemistry images for AchE, BDNF, COX and SOD in hippocampus, which were then quantitatively analyzed by MOD. The positive results of the AchE, BDNF, COX and SOD immunohistochemistry staining showed that brown particles existed in the cytoplasm of the neurons.

The positive particles were distributed densely in cytoplasm of the hippocampus in the control and single 5 mW/cm^2^ groups *(S5, L5)* (Fig. 6). In comparison with the control group, the 5 mW/cm^2^ accumulative exposure *(SL5)* caused significantly reduced expressions of AchE *(p* = *0.025, n* = *5)* and COX *(p* = *0.041, n* = *5)*, the 10 mW/cm^2^ single frequency exposure *(S10, L10)* caused significantly reduced expressions of AchE *(S10, p* = *0.000; L10, p* = *0.000, n* = *5)*, COX *(S10 p* = *0.001; L10, p* = *0.000, n* = *5)* and SOD *(S10, p* = *0.002; L10, p* = *0.001, n* = *5)*, and the 10 mW/cm^2^ accumulative exposure *(SL10)* induced significantly reduced expressions of AchE *(p* = *0.000, n* = *5)*, BDNF *(p* = *0.046, n* = *5)*, COX *(p* = *0.000, n* = *5)* and SOD *(p* = *0.000, n* = *5)*.

#### No frequency-dependent effects between 2.856 GHz and 1.5 GHz microwave in AchE, BDNF, COX and SOD expressions

Based on the quantitative analysis, the results from groups of same power density but different frequency were compared to figure out the frequency-dependent effects *(L5 vs. S5, L10 vs. S10)*. No significant differences were found between 2.856 GHz and 1.5 GHz microwave groups (Fig. 6).

#### Significant does-dependent effects of 2.856 GHz and 1.5 GHz microwave in AchE, COX and SOD expressions

The 10 mW/cm^2^ 2.856 GHz microwave *(S10)* significantly reduced expressions of AchE *(p* = *0.000, n* = *5)*, COX *(p* = *0.018, n* = *5)* and SOD *(p* = *0.018, n* = *5)* when compared with the 5 mW/cm^2^ 2.856 GHz microwave *(S5)*. The 10 mW/cm^2^ 1.5 GHz microwave *(L10)* caused significantly reduced expressions of AchE *(p* = *0.000, n* = *5)*, COX *(p* = *0.012, n* = *5)* and SOD *(p* = *0.002, n* = *5)* compared with the 5 mW/cm^2^ 1.5 GHz microwave *(L5)* (Fig. 6).

#### Possible aggravated interaction effects between 2.856 GHz and 1.5 GHz microwave in AchE expressions

In AchE expressions, significant interactions were found in the 10 mW/cm^2^ accumulative exposure groups *(p* = *0.001, n* = *5)* (Fig. 6).

## Discussion

### The cognitive declines induced by microwave were closely related with the power density

Studies in the last decade suggested that the microwave might have special influences on chemical processes, including the promoting and inhibiting effects^20, 21^. As we all known, biochemical reaction is the nature of human body’s physiological process which happens at every moment. Every system in human bodies depends on the normal and regulated biochemical reaction procession, especially the highly complex nervous system.

Microwave can be absorbed by the biological tissues. The effects caused by absorbed energy can be divided into thermal effects and non-thermal effects ^22^. In this study, we had detected the body surface temperature changes before and immediately after microwave exposure and the changes were less than 1 °C. Parts of microwave energy would be absorbed when passing through the biological body. The living body has abilities to take away the partial heat by blood flow and other physiological regulations. The microwave under this experimental condition did not exceed the range of the organism’s thermoregulation capacity, so the effects in this study were considered to be primarily non-thermal effects.

Changes in behavior were important outcomes for assessing the effects of microwave exposure on cognitive functions^23, 24^. In these studies, microwave could prolong the AELs of rats, which suggested the spatial learning and memory ability was disrupted by microwave exposure^25, 26^. To meet the need of potential hazards prevention, groups with different doses were set up in our study. The 10 mW/cm^2^ microwave caused more serious injuries than the 5 mW/cm^2^ microwave groups. The injury effects were more closely related with the microwave power density.

The electric activities of brain were direct reflections of brain functional statuses and the EEGs were primary tools to detect the dynamic brain functions^27^. In cognitive disorders, the alterations of EEG occurred which conformed with injuries of cognitive *(especially mnemonic)* abilities^28–31^. In our study, according to the power spectral analysis of EEGs, the 2.856 GHz and 1.5 GHz microwave significantly decreased the EEG frequency and power of α, β waves and significantly increased the power of θ, δ waves. According to Thuroczy’s data, local brain exposure of 4 GHz continues wave *(CW)* caused an increase in the power of δ wave^32^. Chizhenkova’s study also exhibited an increase of slow waves in rabbits after 2400 MHz CW radiation^33^. The inhibition of the electrical activity of the brain and decreased in spatial learning and memory ability suggested the hazards effects of 2.856 GHz and 1.5 GHz microwave on nervous system. In our study, there were no significant differences between the 10 mW/cm^2^ groups and the 5 mW/cm^2^ groups. The EEG frequency and power of α, β waves had a decreasing trend with the increase of microwave power, while the power of θ, δ waves had an increasing trend.

The hippocampus was closely related with the learning and memory ability, which was the main area of the limbic system^34^. The results of pathological examination suggested that the 2.856 GHz and 1.5 GHz microwave induced synapse damages as well as mitochondria injuries in hippocampus,which were consistent with the functional study. Injuries in hippocampus were found more serious in the 10 mW/cm^2^ groups than that of the 5 mW/cm^2^ groups.

Based on the morphological results, we hypothesized that the behavioral degeneration and brain electrophysiological disturbances were caused by prominent plasticity lesions and abnormal energy metabolisms. The Nissl substances, rough endoplasmic reticulum (RER) with rosettes of free ribosomes, are important for the protein synthesis^35^. The lost and dissolved of Nissl substances were observed in many neurodegeneration diseases, which were considered as importance factors for cognitive decline^36, 37^. The quantitative analysis showed that the 2.856 GHz and 1.5 GHz microwave significantly reduced the contents of Nissl substances. Moreover, the degrees of reduction was closely related to the power density.

Considering the Nissl substances were of great importance for protein synthesis, the changes in neuronal metabolisms were detected. The expressions of AchE, BDNF, COX and SOD in hippocampus were detected by the immunohistochemistry. AchE was closely related to the metabolism of acetylcholine, which was a kind of neurotransmitter and played an important role in learning and memory^38, 39^. BDNF, a kind of growth factor, was closely related with the synaptic plasticity^40^. COX was the key enzyme in the mitochondrial electron transport chain^41^. SOD was one of the most important antioxygen in biological body^42^. The quantitative analysis showed that all expressions of them declined in varying degrees after S and L band microwave exposure. Our study found that the microwave could affect many metabolic processes in neurons, including oxidative stress, energy metabolism, growth factors and neurotransmitters. We predicted that the general injuries induced by microwave induced the declined cognitive functions. Compared to 5 mW/cm^2^ groups, the contents of AchE, COX and SOD in the 10 mW/cm^2^ groups declined significantly.

Besides, the does-dependent effects were also found in studies on other organs. Liu’s study found that structural damages in the sinoatrial node in rats aggravated with the increase of microwave power^16^. Higher power density groups would have higher SAR values when other conditions were consistent. The SAR values in the 10 mW/cm^2^ groups were much higher than that of 5 mW/cm^2^ groups *(S10: 3.3 W/kg, S5: 1.7 W/kg; L10: 3.7 W/kg, L5: 1.8 W/kg)*. In addition, the exposures of 5 mW/cm^2^ of 1.5~100 GHz microwave for 6 min were described as safe in safe standards (IEEE C95.1).

### The injury effects on cognitive function were similar between 2.856 GHz and 1.5 GHz microwave

As we all known, the physical properties of electromagnetic wave have been closely related with its frequency. The properties of electromagnetic wave are different when the frequency changes. For example, ultraviolet rays could cause ionizing effects while infrared rays mainly caused non-ionizing effects with thermal effects, which was same for microwaves. Properties such as penetrability, carrier ability, reflectivity, and absorptivity were closely related with microwave frequencies.

Microwaves were divided into various bands according to their frequencies. Each band was used for certain applications based on its suitable properties. The frequency-dependent effects on nervous system of microwave should be mentioned.

Nowadays, the does-effect relationship of microwave is attracting many scholars’ attentions, which is very essential for its safety assessment. Therefore, the mostly experimental conditions were microwaves of various power levels but with same frequencies^43, 44^. Therefore, the explorations about frequency-dependent effects were neglected. In this study, the animals were treated with 2 different frequencies: 2.856 GHz and 1.5 GHz. In the cases of identical power density, there were no significant differences of results in the 2.856 GHz groups and the 1.5 GHz groups. The two kinds of microwaves could induce similar results, including prolonged AELs in space navigation tests, fluctuations in EEGs, injuries in morphology and turbulences in various metabolisms.

This was an elementary attempt in the explorations of frequency-dependent effects. The SAR values of the S5 and L5 groups were 1.7 W/kg and 1.8 W/kg respectively. The SAR values of S10 and L10 were 3.3 W/kg and 3.7 W/kg respectively. The SAR values of 2.856 GHz and 1.5 GHz under the same average power density were different, because the radiation parameters were different. However, the SAR values with different frequencies were close. The effects of S5 and L5 or S10 and L10 could be compared.

We believed there would be more findings in the future researches. The frequency-effect relationship might be found in the other bands and frequencies.

### Possible interaction effects were found in accumulative exposure, but the accumulative effect of power cannot be ruled out

Most of the experiments tried to study the effects of certain single frequency microwave exposure, while the interaction effects of multiple frequencies microwaves exposure, especially the 2.856 and 1.5 GHz, were never been discussed.

Some studies aimed at finding the combined effects of cell phone communication signals^10, 45–48^, but they stopped at the hazard evaluation. In those studies, the interaction effects between CDMA and WCDMA were never discussed.

During the cold war, researchers of the superpower focused on the possible military applications of microwave and the ionizing radiation. Michaelson *et al*.^13^ found that the previous exposure of microwave could reduce the mortality of dogs caused by ionizing radiation, while the microwave and ionizing radiation have harmful effects respectively. Those findings suggested the existence of interaction effects of different frequencies electromagnetic waves.

According to the results of statistical analysis, the possible interaction effects were found in in the Nissl substances and AchE expressions. Those findings indicated aggravated interaction effects between 2.856 GHz and 1.5 GHz exposure.

At the same time, the dose-dependent effects indicated the injury effects were dominated by power. According to the SAR values provided (Table 1), the accumulative power in accumulative groups was higher than single frequency exposure groups. The worst injury effects of accumulative exposure could also be explained by the accumulative power. The energy absorbed in accumulative groups was larger than that of single frequency exposure groups.

All in all, the interaction effects between 2.856 GHz and 1.5 GHz were statistically proved, but the cumulative effect of power cannot be ruled out, so we carefully described our findings as possible interaction effects.

### The possible application of findings in this study

In this study, it was demonstrated that the 2.856 GHz and 1.5 GHz microwave could cause generalized injuries in nervous system, including disorders in neurotransmitter, cytokines, oxidative stress and cellular respiration. Data showed that microwave-induced damage was closely related with the molecular mechanism of metabolism. Zuo’s data showed that RKIP might act as a key regulator of neuronal damage caused by microwave exposure^49^. Besides, Zhao *et al*.^50^ found that modulating mitochondrial functions could against microwave-induced injuries in mitochondria, which indicated that there were ways to treat microwave injuries. There were many studies of the drugs for treating microwave-induced injuries, but these drugs were mostly aimed at one type of metabolic or molecular target^51, 52^. Based on our study, it was not enough to cure the microwave-induced injuries by unilateral therapy because of the generalized effects caused by microwaves on the organism.

Our study found that the microwave had damaging effects on the neuron structure, which indicated that microwave could be used to destroy the nervous system. At present, the clinical microwave ablation therapy requires the microwave antenna be implanted into the target area, which is an invasive method^53^. Therefore, aggravated injuries in accumulative exposure were noteworthy, which might make the non-invasive microwave ablation possible.

If the organism is considered as a homogeneous object, multiple beams of certain interactive frequency microwaves can converge exactly at one point and cause aggravating interaction effects. The tissue at that point would be destroyed while the surrounding tissue was safe. This provided a new idea for noninvasive radiofrequency treatment. However, the structure of the organism is not homogeneous, how to avoid the linear propagation of the microwave disturbed by the complex structure and to achieve accurate focus is still a problem.

### Unresolved issues

Although electromagnetic wave technologies have brought a lot of conveniences for people’s lives, the understanding of electromagnetic waves was still far from enough. The field of bio-electromagnetics still stayed on the level of biological effect explorations. At molecular level, the interactions between electromagnetic waves and biological macromolecules were not clear. The physical mechanisms of microwave-mediated chemical reaction were unknown. In the process of multi-disciplinary integration, advances in medicine needed the progresses of biophysics.

## References

[1] Kesari KK, Siddiqui MH, Meena R, Verma HN, Kumar S (2013). Cell phone radiation exposure on brain and associated biological systems. Indian journal of experimental biology.

[2] Szmigielski S (2013). Reaction of the immune system to low-level RF/MW exposures. Sci Total Environ.

[3] Wang H (2015). The relationship between NMDA receptors and microwave-induced learning and memory impairment: a long-term observation on Wistar rats. International journal of radiation biology.

[4] Baan R (2011). Carcinogenicity of radiofrequency electromagnetic fields. The Lancet. Oncology.

[5] Wang H (2013). Impairment of long-term potentiation induction is essential for the disruption of spatial memory after microwave exposure. International journal of radiation biology.

[6] Wang LF (2015). Microwave-Induced Structural and Functional Injury of Hippocampal and PC12 Cells Is Accompanied by Abnormal Changes in the NMDAR-PSD95-CaMKII Pathway. Pathobiology: journal of immunopathology, molecular and cellular biology.

[7] Orendacova J, Orendac M, Racekova E, Marsala J (2007). Neurobiological effects of microwave exposure: a review focused on morphological findings in experimental animals. Archives italiennes de biologie.

[8] Qiao S (2014). Reduction of phosphorylated synapsin I (ser-553) leads to spatial memory impairment by attenuating GABA release after microwave exposure in Wistar rats. PloS one.

[9] Kubinyi G (1996). Effect of continuous-wave and amplitude-modulated 2.45 GHz microwave radiation on the liver and brain aminoacyl-transfer RNA synthetases of in utero exposed mice. Bioelectromagnetics.

[10] Jin YB (2013). Effects of simultaneous combined exposure to CDMA and WCDMA electromagnetic fields on serum hormone levels in rats. Journal of radiation research.

[11] Jin YB (2011). One-year, simultaneous combined exposure of CDMA and WCDMA radiofrequency electromagnetic fields to rats. Int J Radiat Biol.

[12] Jin YB (2012). Effects of simultaneous combined exposure to CDMA and WCDMA electromagnetic field on immune functions in rats. Int J Radiat Biol.

[13] Michaelson, S., Thomson, R. & Quinlan, W. Tolerance of dogs to microwave exposure under various conditions. *Ind Med Surg*, 298 (1961).

[14] Michaelson, S., Thomson, R. & Odland, L. The effects of microwaves on the response to ionizing radiation. *Aerospace Med*, 345 (1962). 13935444

[15] Tikhonchuk, V., Ushakov, I. & Fedorov, V. Structural and metabolic analysis of the reaction of the central nervous system to the combined action of microwave and ionizing radiations. *Radiobiogiia* (1987). 3615818

[16] Liu YQ (2015). Pathological changes in the sinoatrial node tissues of rats caused by pulsed microwave exposure. Biomed Environ Sci.

[17] Wu T (2010). Whole-body new-born and young rats’ exposure assessment in a reverberating chamber operating at 2.4 GHz. Physics in medicine and biology.

[18] Esmekaya MA, Seyhan N, Omeroglu S (2010). Pulse modulated 900 MHz radiation induces hypothyroidism and apoptosis in thyroid cells: a light, electron microscopy and immunohistochemical study. International journal of radiation biology.

[19] Ellis EA (2014). Staining sectioned biological specimens for transmission electron microscopy: conventional and en bloc stains. Methods in molecular biology.

[20] Chen PK, Rosana MR, Dudley GB, Stiegman AE (2014). Parameters affecting the microwave-specific acceleration of a chemical reaction. The Journal of organic chemistry.

[21] Zhou J (2016). A new type of power energy for accelerating chemical reactions: the nature of a microwave-driving force for accelerating chemical reactions. Scientific reports.

[22] Rougier C, Prorot A, Chazal P, Leveque P, Leprat P (2014). Thermal and nonthermal effects of discontinuous microwave exposure (2.45 gigahertz) on the cell membrane of Escherichia coli. Applied and environmental microbiology.

[23] Narayanan SN, Kumar RS, Potu BK, Nayak S, Mailankot M (2009). Spatial memory performance of Wistar rats exposed to mobile phone. Clinics.

[24] Ning W (2007). Effects of GSM 1800 MHz on dendritic development of cultured hippocampal neurons. Acta pharmacologica Sinica.

[25] Lu Y (2012). Glucose administration attenuates spatial memory deficits induced by chronic low-power-density microwave exposure. Physiology & behavior.

[26] Zhao L (2012). Relationship between cognition function and hippocampus structure after long-term microwave exposure. Biomedical and environmental sciences: BES.

[27] Srinivasan R (2006). Anatomical constraints on source models for high-resolution EEG and MEG derived from MRI. Technology in cancer research & treatment.

[28] Dringenberg HC (2000). Alzheimer’s disease: more than a ‘cholinergic disorder’ - evidence that cholinergic-monoaminergic interactions contribute to EEG slowing and dementia. Behavioural brain research.

[29] Dringenberg HC, Diavolitsis P, Noseworthy PA (2000). Effect of tacrine on EEG slowing in the rat: enhancement by concurrent monoamine therapy. Neurobiology of aging.

[30] Penttila M, Partanen JV, Soininen H, Riekkinen PJ (1985). Quantitative analysis of occipital EEG in different stages of Alzheimer’s disease. Electroencephalography and clinical neurophysiology.

[31] Vanderwolf CH (1988). Cerebral activity and behavior: control by central cholinergic and serotonergic systems. International review of neurobiology.

[32] Thuroczy G, Kubinyi G, Bodo M, Bakos J, Szabo LD (1994). Simultaneous response of brain electrical activity (EEG) and cerebral circulation (REG) to microwave exposure in rats. Reviews on environmental health.

[33] Chizhenkova RA (1988). Slow potentials and spike unit activity of the cerebral cortex of rabbits exposed to microwaves. Bioelectromagnetics.

[34] Schmidt B, Redish AD (2013). Neuroscience: Navigation with a cognitive map. Nature.

[35] Beaudet A, Rambourg A (1983). The tridimensional structure of Nissl bodies: a stereoscopic study in ventral horn cells of rat spinal cord. The Anatomical record.

[36] Li J, Wen PY, Li WW, Zhou J (2015). Upregulation effects of Tanshinone IIA on the expressions of NeuN, Nissl body, and IkappaB and downregulation effects on the expressions of GFAP and NF-kappaB in the brain tissues of rat models of Alzheimer’s disease. Neuroreport.

[37] Dziewulska D, Gogol A, Gogol P, Rafalowska J (2013). Enlargement of the Nissl substance as a manifestation of early damage to spinal cord motoneurons in amyotrophic lateral sclerosis. Clinical neuropathology.

[38] Chtourou Y, Gargouri B, Kebieche M, Fetoui H (2015). Naringin Abrogates Cisplatin-Induced Cognitive Deficits and Cholinergic Dysfunction Through the Down-Regulation of AChE Expression and iNOS Signaling Pathways in Hippocampus of Aged Rats. Journal of molecular neuroscience: MN.

[39] Nowakowska E, Chodera A, Kus K, Nowak P, Szkilnik R (2001). Reversal of stress-induced memory changes by moclobemide: the role of neurotransmitters. Polish journal of pharmacology.

[40] Wang Y, Liu H, Zhang BS, Soares JC, Zhang XY (2016). Low BDNF is associated with cognitive impairments in patients with Parkinson’s disease. Parkinsonism & related disorders.

[41] Gu YL (2014). Cognitive improvement of mice induced by exercise prior to traumatic brain injury is associated with cytochrome c oxidase. Neuroscience letters.

[42] Talarowska M (2014). Manganese superoxide dismutase gene expression and cognitive functions in recurrent depressive disorder. Neuropsychobiology.

[43] Shahin S (2013). 2.45 GHz microwave irradiation-induced oxidative stress affects implantation or pregnancy in mice, Mus musculus. Applied biochemistry and biotechnology.

[44] Wang C (2015). Effects of pulsed 2.856 GHz microwave exposure on BM-MSCs isolated from C57BL/6 mice. PloS one.

[45] Kim HN (2012). Analysis of the cellular stress response in MCF10A cells exposed to combined radio frequency radiation. Journal of radiation research.

[46] Lee HJ (2012). The effects of simultaneous combined exposure to CDMA and WCDMA electromagnetic fields on rat testicular function. Bioelectromagnetics.

[47] Lee HJ (2011). Lymphoma development of simultaneously combined exposure to two radiofrequency signals in AKR/J mice. Bioelectromagnetics.

[48] Lee HJ (2009). Lack of teratogenicity after combined exposure of pregnant mice to CDMA and WCDMA radiofrequency electromagnetic fields. Radiation research.

[49] Zuo H (2015). RKIP Regulates Neural Cell Apoptosis Induced by Exposure to Microwave Radiation Partly Through the MEK/ERK/CREB Pathway. Molecular neurobiology.

[50] Zhao L (2014). Upregulation of HIF-1alpha via activation of ERK and PI3K pathway mediated protective response to microwave-induced mitochondrial injury in neuron-like cells. Mol Neurobiol.

[51] Zhang J (2014). AduoLa Fuzhenglin down-regulates microwave-induced expression of beta1-adrenergic receptor and muscarinic type 2 acetylcholine receptor in myocardial cells of rats. Biomedical and environmental sciences: BES.

[52] Zhang X (2014). The compound Chinese medicine “Kang Fu Ling” protects against high power microwave-induced myocardial injury. PLoS One.

[53] Carberry, G. A. *et al*. Pulmonary Microwave Ablation Near the Heart: Antenna Positioning Can Mitigate Cardiac Complications in a Porcine Model. *Radiology*, 160831, doi:10.1148/radiol.2016160831 (2016). 10.1148/radiol.2016160831 PMC5330302 27732159

